# Dietary Gluten and Neurodegeneration: A Case for Preclinical Studies

**DOI:** 10.3390/ijms21155407

**Published:** 2020-07-29

**Authors:** Mahesh Mohan, Chioma M. Okeoma, Karol Sestak

**Affiliations:** 1 Texas Biomedical Research Institute, Southwest National Primate Research Center, San Antonio, TX 78227, USA; 2 Department of Pharmacology, Renaissance School of Medicine, Stony Brook University, Stony Brook, NY, 11794-8651, USA; chioma.okeoma@stonybrook.edu; 3 Tulane National Primate Research Center, Covington, LA 70433, USA; 4 PreCliniTria, LLC., Mandeville, LA 70471, USA

**Keywords:** gluten, celiac disease, neurodegeneration, tissue transglutaminase 2 and 6, rhesus macaque, *OCLN*, *PPARγ*, cannabinoids, dysbiosis, biomolecular condensates, extracellular vesicles

## Abstract

Although celiac disease (CD) is an autoimmune disease that primarily involves the intestinal tract, mounting evidence suggests that a sizeable number of patients exhibit neurological deficits. About 40% of the celiac patients with neurological manifestations have circulating antibodies against neural tissue transglutaminase-6 (tTG6). While early diagnosis and strict adherence to a gluten-free diet (GFD) have been recommended to prevent neurological dysfunction, better therapeutic strategies are needed to improve the overall quality of life. Dysregulation of the microbiota-gut-brain axis, presence of anti-tTG6 antibodies, and epigenetic mechanisms have been implicated in the pathogenesis. It is also possible that circulating or gut-derived extracellular structures and including biomolecular condensates and extracellular vesicles contribute to disease pathogenesis. There are several avenues for shaping the dysregulated gut homeostasis in individuals with CD, non-celiac gluten sensitivity (NCGS) and/or neurodegeneration. In addition to GFD and probiotics, nutraceuticals, such as phyto and synthetic cannabinoids, represent a new approach that could shape the host microbiome towards better prognostic outcomes. Finally, we provide a data-driven rationale for potential future pre-clinical research involving non-human primates (NHPs) to investigate the effect of nutraceuticals, such as phyto and synthetic cannabinoids, either alone or in combination with GFD to prevent/mitigate dietary gluten-induced neurodegeneration.

## 1. Introduction

As early as in 1908, the first reports of neurological abnormalities and complications in patients with gastrointestinal “sprue”, such as peripheral neuritis, ataxia, and partial degeneration of spinal cord, started to emerge [1]. The first systematically corroborated evidence of dietary gluten-associated (celiac) neuropathy dates back to 1966, when Cooke and colleagues [2] described neurodegenerative lesions in muscle biopsies from adult patients with celiac disease (CD) using a combination of electron microscopy and vital (H&E) staining. Thereafter, in 1962, the first fatal case resulting from neurological disease was reported in a 57-year old patient with CD [3]. The postmortem findings in this patient were compared with nine other celiac cases that also exhibited progressive central nervous system (CNS) disorders. It was concluded that progressive neurodegeneration in the cerebellum, deep gray matter, brain stem, and spinal cord were the common histopathological characteristics in these patients [3]. Another report, involving a 47-year old CD patient with spinocerebellar degeneration and normal vitamin E levels, revealed that gluten-free diet (GFD) administered to celiac patients can have beneficial effects in stabilizing not only gastrointestinal, but also neurological symptoms [4]. Epidemiological survey of people living on the remote Micronesian islands consuming traditional grain-free versus western-type (grain-rich) diet showed that the latter group suffered with significantly higher incidence of schizophrenia [5], a neurodevelopmental but also a progressively neurodegenerative disorder [6,7]. Nonetheless, many of the initial reports pointing to an association between neurological disorders and consumption of gluten-containing diet lacked direct evidence and in-depth analysis. 

The landscape has started to change in recent decades after a large body of evidence regarding the role of tissue transglutaminases (tTGs), namely the intestinal tissue transglutaminase-2 (tTG2), emerged with respect to CD pathogenesis [8]. It was established that in addition to post-translational modification of gluten/gliadin through the process of deamidation/transamidation, tTG2 functions as an autoantigen, thereby inducing the formation of tTG2 autoantibodies. Presence of tTG2 autoantibodies occurs in almost 100% of celiac patients but only in a small fraction of non-celiac gluten sensitivity (NCGS) patients [9]. Involvement of the neural isoform of tTG, i.e., tTG6 was suggested in the pathogenesis of several neurodegenerative disorders including Alzheimer’s Disease (AD) and Huntington’s Disease (HD), where tTG6 was reported to contribute to the resolution of abnormally accumulated amyloid and microtubule-associated proteins [10]. In addition, tTG6 was shown to be implicated in the pathogenesis of movement disorders, such as gluten ataxia and multiple sclerosis (MS) [11,12,13,14]. Increased levels of circulating anti-tTG6 antibodies were also detected in adult patients with schizophrenia [15]. Finally, recent human leukocyte antigen (HLA)-typing of CD and NCGS patients diagnosed with Autism Spectrum Disorders (ASD) and/or Down’s Syndrome (DS), demonstrated that there is a definitive, and significant association between specific HLA haplotypes and dysregulation of the gut-brain axis [16,17,18]. 

In people with psychosis and multi-episode schizophrenia, it was shown that significant proportion of these individuals have elevated anti-gliadin antibodies but not necessarily antibodies to deamidated gliadin and tTG (autoantibodies), confirming that neuro-immune responses to gliadin differ between CD and NCGS [19]. On the other hand, due to relatively low CD predictive value of anti-gliadin antibodies (~70–80%), their presence might be even less/not predictive in patients with neurodegeneration. Furthermore, antibodies to glutamic acid decarboxylase (GAD), a protein important in the synthesis of inhibitory neurotransmitter gamma-aminobutyric acid, were found to be present in gluten-sensitive people with neurological disorders, but not in CD patients [20]. In addition to autoantibodies recognizing the neural tTG6 in celiac patients with neurological disorders, autoantibodies reactive to synapsin I (neuronal synaptic vesicles), neuroglial, and Purkinje cells were identified [21,22,23]. 

Taken together, it became evident that dietary gluten-induced neurodegeneration is a serious and understudied phenomenon, the pathogenesis of which differs between CD and NCGS. TG6 has been proposed to play an important role in the neurological (both central and peripheral nervous system) manifestations of CD, as high TG6 antibody levels have been detected in CD patients with gluten ataxia [12] and peripheral neuropathy [24]. Due to the absence of tTG2/6 involvement in NCGS patients, it was proposed that a leaky intestinal epithelial barrier that allows partially digested immunotoxic gluten peptides to enter the systemic circulation and then cross the blood brain barrier, where these peptides along with HLA-DQ2/DQ8 restricted CD4 T cells infiltrating the brain from the intestine may produce proinflammatory cytokines driving CNS disease in NCGS patients [25,26,27,28]. Alternatively, circulating or gut derived extracellular structures, including biomolecular condensates and extracellular vesicles from CD and NCGS patients may use a leaky intestinal epithelial barrier to deliver their proinflammatory cargo to the brain. 

## 2. Results

### 2.1. Preclinical Evidence

Currently, there is limited preclinical evidence to demonstrate the link between gluten and neurodegeneration. Despite attempts to develop gluten ataxia in laboratory mice, such efforts were limited or not successful [29,30]. 

**Figure 1:**
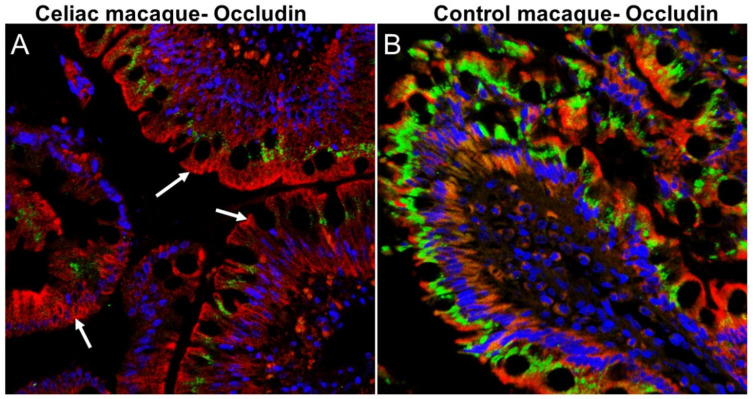
Occludin (OCLN) protein expression is significantly decreased in duodenal epithelium of celiac macaques. All panels involve triple labels with OCLN (**green**), cytokeratin (**red**) and Topro3 for nuclear staining (**blue**). Colocalization appears yellow. Note the marked loss of occludin protein (white arrow) staining in the duodenal epithelium (DE) of the celiac macaque (**A**). In contrast, occludin protein (**B**) staining is intense in the DE of the control macaque. Magnification for both panels is 40×. Occludin (Cat#LS-B2437) antibody that cross reacts with the rhesus macaque was purchased from Lifespan Biosciences, Seattle, WA, USA.

In studies using the experimentally-induced rhesus macaque model of CD, i.e., gluten-sensitive enteropathy (GSE), it was demonstrated that several tight junction and their associated proteins, such as zonulin and haptoglobin-2, known to be expressed also in human blood brain barrier, were dysregulated in celiac rhesus macaques [31]. In CD patients, reduced expression of intestinal tight junction proteins including zonulin and occludin were linked to increased epithelial permeability, i.e., leaky gut [32]. Further, zonulin receptor has been identified as the precursor for haptoglobin-2 [28]. Similarly, we also reported significant downregulation of intestinal tight junction proteins zona occludens-1 (ZO1) and claudin-1 in duodenum of celiac macaques [32]. In recent studies, we detected markedly reduced occludin protein expression in the duodenal epithelium of celiac macaques (Figure 1). A complete loss of occludin protein expression from the apical and basal side of the duodenal epithelium of celiac macaques was noted (Figure 1A) while its high basal expression was present in healthy control macaques (Figure 1B). Evidence from studies involving psychotic patients confirmed increased levels of serum haptoglobin-2, consistent with findings from celiac macaques [28,31]. Dysregulated and impaired expression of zonulin in celiac macaques suggests that both zonulin and haptoglobin-2 could negatively impact the functioning of the blood brain barrier [28,31]. Finally, a group of dysregulated genes associated with perturbed neurological functions were also identified in celiac macaques fed a gluten-containing diet [33]. The predisposition genes potentially linked to the neurological form of CD were identified by messenger-RNA (mRNA) profiling: *CADPS2* (ASD), *CAPN13* (AD), *BACE2* (AD, DS), *DSCR5* (DS), and *PINK1* (Parkinson’s Disease (PD) [33]. Expression levels of these mRNAs were not perturbed in healthy macaques and were only minimally so in celiac macaques on GFD, suggesting that consumption of dietary gluten in susceptible primates is linked, besides other effects, to neurological dysfunction. To corroborate and to further expand these findings, more translational studies employing the rhesus macaque celiac model are needed. 

### 2.2. Mechanisms of Dietary Gluten-Induced Neuropathy

It was established that increased tTG2 activity leads to autoimmune reaction and GSE, i.e., CD in genetically-predisposed individuals [34,35,36]. Besides gluten digestion, tTG-mediated glutamine deamidation can, in some celiac patients, lead to the aggregation of cerebral β-amyloid, one of the hallmarks of neurodegeneration in people with PD, HD, and AD [37,38,39,40,41,42]. It is not clear however, if neuronal dysfunction occurs in all individuals with CD or if this is limited only to a subset of these patients. Due to tTG’s capability to be i) recognized as autoantigen in not only intestinal but also systemic tissues including CNS, ii) to cause cerebral β-amyloid polymerization, and iii) to facilitate inflammation and cancer, it became an attractive drug target for a multitude of diseases [36,37,39,43]. A substantial number of inhibitors, probes, and substrates were chemically engineered with the purpose to better understand the pathogenesis of CD and to use some of the tTG inhibitors as therapeutics [44,45]. Notwithstanding, in vivo use of these compounds in the treatment of tTG-associated illnesses is not straight-forward and requires thorough translational validation using a model that faithfully recapitulates human disease. Moreover, the contribution of dysbiotic microbial metabolome to post-translational modifications of CD-relevant proteins, such as tTGs, was suggested to influence functioning of the gut-brain axis [46,47].

### 2.3. MicroRNA Evidence

The evaluation of the role of micro-RNAs (miRNAs) is of great interest in CD as they represent an important epigenetic mechanism with immense potential to regulate the inflammatory response associated with CD pathogenesis. MiRNAs are ~20–23 nucleotide long, small RNA molecules that regulate gene expression post-transcriptionally by binding to homologous sequences on the 3’ untranslated regions (UTRs) (homologous base pairings between miRNA seed nucleotides 2 to 7 and the 3’ UTR). MiRNAs are known to regulate majority of cellular processes that include but are not limited to cell proliferation, differentiation, apoptosis, cell signaling, immune, and inflammatory responses. Over the past decade, the role of miRNAs in CD pathogenesis has been studied in immune cells isolated from intestinal biopsies and peripheral blood. Using duodenal pinch biopsies, Magni and colleagues [48] identified significant downregulation of miR-192-5p, miR-31-5p, miR-338-3p, and miR-197 in patients with celiac disease with severe histopathological lesions. Consistent with miR-192-5p downregulation, several bioinformatically predicted targets with critical roles in innate immune response, namely, chemokine C-X-C motif ligand 2 (*CXCL2*) and nucleotide oligomerization domain-2 (*NOD2*) showed marked upregulation at both the mRNA and protein level. In a separate study, Vaira and colleagues [49] further confirmed inflammation induced downregulation of miR-192-5p. Interestingly, miR-192-5p is also downregulated in patients with ulcerative colitis, which suggests that it plays a crucial role in maintaining intestinal homeostasis [50]. In addition, forkhead box P3 (*FOXP3*), Run-related transcription factor 1, and interleukin-18 which are predicted targets of miR-31-5p, miR-338-3p, and miR-197 also showed significantly high expression in biopsy tissues. More recently, expression of miR-192/215 together with the miR-200 families were shown to be progressively reduced and those of miR-17/92 and C19MC miRNAs to be upregulated in refractory CD and intestinal T-cell lymphomas associated with CD [51]. A link between downregulation of these miRNAs resulting in constitutive signal transducer and activator of transcription 3 (*STAT3*) activation and MYC proto-oncogene BHLH transcription factor (*c-Myc*) mediated oncogenic signaling in refractory CD and subsequent lymphomagenesis was proposed [51]. 

Unlike miR-192-5p, in pediatric CD, miR-449a expression was significantly increased [52]. Notch receptor 1 (*NOTCH1*) and kruppel like factor 4 (*KLF4*) that are associated with goblet cell proliferation and differentiation was validated as direct targets of miR-449a. Consistent with miR-449a mediated negative regulation of *NOTCH1* and *KLF4*, goblet cell numbers were markedly reduced in the small intestines of children with CD. Interestingly, miR-21 and miR-31 have also been identified as circulating non-invasive biomarkers of pediatric CD [53]. Increased miR-21 and decreased miR-31 was confirmed in serum of pediatric CD patients. Additionally, miR-21 correlated with the presence of IgA-tTG2 autoantibodies. 

**Figure 2:**
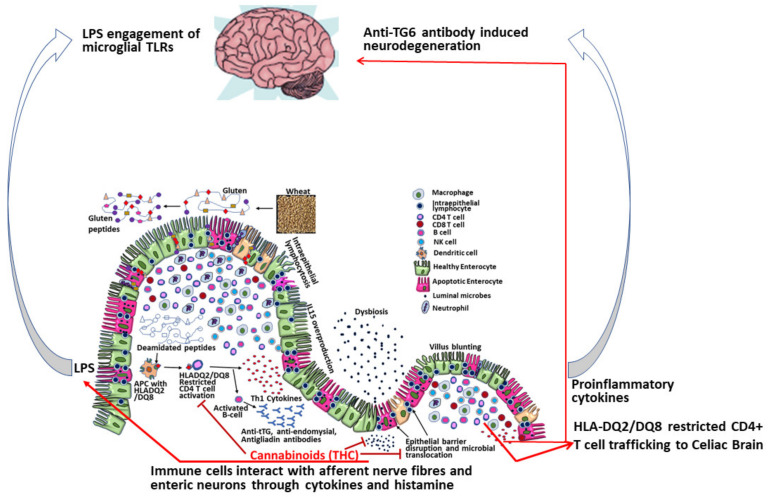
Immunopathology of central nervous system (CNS) disease in celiac disease patients and potential beneficial (treatment) role of phytocannabinoids as nutraceuticals to mitigate gluten induced intestinal and CNS inflammation. THC—delta-9-tetrahydrocannabinol; LPS—lipopolysaccharide; TG6—transglutaminase-6; TLR—Toll-like receptor; APC—antigen presenting cell.

Apart from targeting innate immune response genes, our own studies using the rhesus macaque model of GSE confirmed elevated miR-204 to directly target the intestinal tight junction protein claudin-1 resulting in its significantly reduced protein expression in the duodenum of celiac macaques [32]. Further, intestinal inflammation coupled with barrier disruption was accompanied by marked gut dysbiosis [32]. Disruption of the intestinal barrier can facilitate translocation of intestinal microbes and microbial by-products into the systemic circulation. In longstanding cases of CD, prolonged microbial translocation can exceed hepatic clearance thereby increasing the potential for lipopolysaccharide (LPS) to accumulate, cross the blood brain barrier and activate microglia cells in the CNS resulting in neuroinflammation and neuronal damage (Figure 2). Overall, these findings identify a putative miRNA mediated mechanism that can facilitate intestinal inflammation and eventually trigger neurological injury in CD patients. Interestingly, our previously published studies using the simian immunodeficiency virus (SIV)-infected rhesus macaque model of acquired immune deficiency syndrome (AIDS) demonstrated the ability of phytocannabinoids to inhibit intestinal inflammation by inducing the expression of anti-inflammatory miRNAs, inhibiting T cell proliferation/activation and preserving intestinal tight junction protein expression [54,55]. These latter findings accentuate the potential of cannabinoids as a viable nutraceutical either alone or in combination with other immunobiologics to reduce dietary gluten-induced intestinal inflammation, epithelial barrier disruption, and extraintestinal complications including neurological disease in CD patients (Figure 2). 

### 2.4. Gut Dysbiosis-Neurodegeneration Link

**Figure 3:**
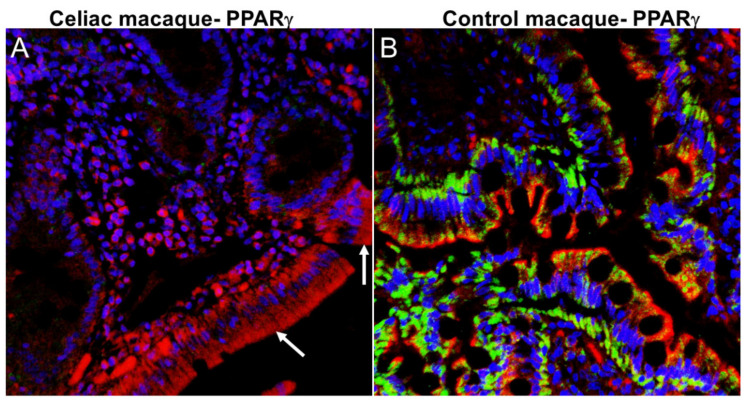
Peroxisome proliferator activator receptor gamma (*PPARγ*) protein expression is significantly decreased in DE of celiac macaques. All panels involve triple labels with *PPAR*γ (**green**), cytokeratin (**red**) and Topro3 for nuclear staining (**blue**). Colocalization appears yellow. Note the marked loss *PPARγ* (**A**) (white arrow) staining in the DE of the celiac macaque. In contrast, PPARγ protein (**B**) staining is intense in the DE of the control macaque. Magnification for both panels is 40×. *PPARγ* (Cat#LS-B651-50) antibody that cross reacts with the rhesus macaque was purchased from Lifespan Biosciences, Seattle, WA, USA.

It was postulated that homeostasis of the gut-brain axis can be disrupted in association with progressed age, obesity, diet, and drug use [26]. Chronic intestinal inflammation that is triggered in predisposed (CD/NCGS) individuals by consumption of gluten-containing diet is intimately linked with intestinal dysbiosis and leaky gut [32,54]. We and others have demonstrated that dietary gluten-induced inflammation and dysbiosis are linked with perturbations of genetic regulatory factors of neuroinflammation, cognition, and neurodegeneration [33,56,57]. From a molecular mechanistic perspective, the expression of peroxisome proliferator activated receptor gamma (*PPARγ*), a key gene with anti-inflammatory (peripheral, intestinal and neuroinflammation) [58] and anti-dysbiotic effects [59] is considerably reduced in ulcerative colitis [60] and celiac disease [61,62,63] patients. Byndloss et al. [59] demonstrated that *PPARγ* downregulation was associated with dysbiotic expansion of bacteria belonging to the family *Enterobacteriaceae* (phylum Proteobacteria) and reduction in the relative abundance of obligate anerobic bacteria. Like celiac patients, we detected markedly reduced *PPARγ* expression in duodenal epithelium of celiac macaques (Figure 3A) relative to healthy control macaques (Figure 3B). Similar to occludin protein expression (Figure 1A), considerable loss of *PPARγ* protein expression from the duodenal epithelium was detected in celiac macaques. Accordingly, *PPARγ* downregulation may promote intestinal inflammation and subsequent dysbiosis in celiac macaques and by extension in patients with CD. 

It would be an oversimplification to suggest that gut dysbiosis-associated neuropathology can be resolved by a strict adherence to GFD. Nevertheless, research regarding the inflammatory pathways and microbial taxa in affected individuals is already paving the way for the formulation of new prevention and treatment strategies [32]. Accordingly, it was suggested that remission of dysbiosis in individuals with autism and other neurodevelopmental disorders could be the first step in the treatment of these illnesses [64]. Although gluten-free diets (GFD) have shown success in restoration of *PPARγ* expression in celiac patients [62], the risks of gluten contamination coupled with the persistence of intestinal inflammation in patients with CD on GFD underscores the need to develop better therapeutic solutions. Accordingly, reducing chronic inflammation, promoting intestinal mucosal healing and repair, and restoring epithelial barrier integrity and the microbiome are the topmost priorities of the celiac research community. Because *PPARγ* was described to be the key functional receptor transducing the effects of commonly prescribed anti-inflammatory amino salicylates in inflammatory bowel disease (IBD) patients [65], augmenting *PPARγ* expression represents a promising approach for the clinical management of gluten induced intestinal inflammation [66]. In this context, mesalazine also known as 5-amino salicylic acid (5-ASA) was shown to reduce oxidative burst and induce *PPARγ* expression in ex vivo cultures of duodenal biopsies obtained from newly diagnosed celiac patients [66]. In addition, mesalazine treatment also reduced protein levels of nuclear factor kappa B (NFκB) and nitric oxide synthase 2 (NOS2), an enzyme that produces nitric oxide [66]. Obligate anerobic bacteria have been shown to activate PPARγ signaling to limit the production of nitrate and oxygen electron acceptors by host epithelial cells, thereby preventing the expansion of facultative anerobic bacteria belonging to the family *Enterobacteriaceae* (phylum Proteobacteria) [59]. Therefore, reduced PPARγ signaling in celiac patients and macaques may promote the activation of NOS2, resulting in increased production of nitric oxide, which can then be broken down to non-toxic nitrates in the intestinal lumen and made abundantly available as a respiratory electron acceptor to members of the *Enterobacteriaceae* family that encode nitrate reductases. Although 5-ASA has been effective, it also has produced adverse effects [67], which makes it critical to develop newer approaches to restore and activate PPARγ signaling to reduce intestinal dysbiosis and prevent microbial products from reaching the brain and activate neuroinflammatory signaling. Moreover, in this context, we recently demonstrated the ability of cannabinoids to directly trans activate *PPARγ* expression in in vitro cultured cell lines and in ex vivo colon explant cultures [68]. Most notably, using the SIV-infected rhesus macaque model of AIDS, we found that long-term controlled low dose administration of naturally available phytocannabinoids like delta-9-tetrahydrocannabinol (THC) not only inhibited intestinal inflammation [55] but also inflammation of the minor salivary glands in the oral cavity [69]. Specifically, THC treated chronically SIV-infected rhesus macaques had better preservation of oral commensal bacteria species that are critical to the maintenance of oral microbial homeostasis (Mohan personal communication). In addition, chronic THC significantly reduced the relative abundance of pathogenic bacteria associated with periodontitis (Mohan personal communication). These findings are exciting and promising as it represents a safe yet pharmacologically feasible new approach to inhibit intestinal and neuroinflammation, restore epithelial barrier integrity, and prevent dysbiosis in not only celiac, but also other chronic inflammatory diseases of the intestine.

### 2.5. Potential Role of Extracellular Structures—Biomolecular Condensates and Extracellular Vesicles in Pathogenesis of CD-Associated Neurodegeneration

It is becoming increasingly evident that all cells create discrete biochemical environments in the form of extracellular structures (ES), used to organize and orchestrate complex biochemical reactions that regulate host health and disease. For the purpose of this review, ES are defined as non-replicative organism-derived acellular structures that consist of two archetypes—namely, i) the membrane-less biomolecular condensates (BMCs), and ii) the membrane-encased extracellular vesicles (EVs). While BMCs assemble by thermodynamic-mediated liquid-liquid phase separation, the liquid BMCs are known to transform into reversible amyloid fibers or insoluble aggregates [70]. While the details of BMC biology is still evolving, it has been shown that RNA and proteins with intrinsically disordered regions are key factors that influence the intracellular composition, size, and morphology of BMCs [71,72]. Unlike BMCs, EVs contain at least one membrane. EVs are comprised of at least three different subtypes, including apoptotic bodies, released as a product of apoptotic cell disassembly; microvesicles, generated by the outward budding and fission of membrane vesicles from the cell surface; and exosomes, generated by reverse budding of multivesicular bodies [73,74]. These EV subtypes are characterized by their size, although size range overlaps exist. Apoptotic bodies are generally larger (500–4000 nm) in size, microvesicles usually range in size from 50–2000 nm, most being larger than 200 nm, and exosomes from 30 to 100 nm [75,76,77]. Despite the size range, different cells, tissues, and organisms release vesicles that vary in size. Another characteristic of EVs is their ability to encase bioactive cargo that includes subsets of diverse proteins, different RNA species (mRNAs, miRNA, tRNA, etc), dsDNA, extrachromosomal DNA, lipids, pathogen-derived products, and illicit and licit drugs [78,79,80]. Given the diverse nature of EVs and the overlap in their size and cargo composition, we will use the term EVs to encompass apoptotic bodies, microvesicles, and exosomes. Both BMCs and EVs regulate host response to disease, immunological, and environmental assaults. Hence, BMCs play important roles in various physiological contexts, such as transcription and stress response [81,82], and in pathophysiological conditions, including cancer and neurodegenerative diseases [83,84,85]. EVs mediate distal and proximal intercellular communications [86,87,88] and organism-to-cell interactions [89,90,91], and are involved in neuroinflammation [92], irritable bowel syndrome (IBS) [93], and, potentially, CD [94]. 

### 2.6. ES and Neurodegeneration

ES—BMCs and EVs display both beneficial and detrimental roles in various disease contexts. With regards to the CNS that relies on complex cross talk amongst CNS cellular and acellular components, it has been shown that BMCs and EVs display both anti- and pro- inflammatory responses in inflammation-associated neurodegenerative diseases, such as AD, frontotemporal dementia (FTD), and amyotrophic lateral sclerosis (ALS), HD, and PD. It is undisputable that neurodegenerative disorders involve BMCs, in the form of regional aggregation of cytosolic or nuclear proteins, which are believed to drive neurodegeneration [95]. Examples of the neuro-associated BMCs include the proteinaceous assemblies of the microtubule-associated protein Tau (MAPT), cytosolic inclusions of the RNA-binding proteins TAR DNA binding protein of 43 kDa (TDP-43), fused in Sarcoma (FUS), and poly-glutamine (polyQ) aggregates [96]. Tau aggregates are mostly found in AD, TDP-43 and FUS are found in ALS and FTD, while polyQ aggregates more in HD [96]. Interestingly, BMC aggregates are thought to spread from one brain region to another, in line with the progressive nature of these diseases [97]. Similar to BMCs, EVs have been implicated in the pathogenesis and these membrane structures are integral component of neuroimmune interaction. For example, CNS-derived EVs are present in peripheral circulation [95,96]. The CNS EVs play a role in assessing cerebral neuroimmune status. For example, it is known that the initiation, propagation, and resolution of inflammatory response to CNS injury or neurodegenerative disease relies on cytokines, miRNAs, or microbial products, all of which have been shown to be present in EVs [79,98,99,100,101,102]. Additionally, EVs mediate the transfer of peripheral inflammatory molecules to the CNS [103]. Although BMCs and EVs are different in structure and function, there seem to be convergence in their function. Both BMCs and EVs have been implicated in contributing to the pathogenesis of neurodegenerative diseases where the pathology is dictated in part by neuroimmune mechanisms, such as the presence of amyloid-β peptide (Aβ) [104,105,106] and Tau [95,107].

### 2.7. ES and the Gut-Brain Barrier

Although the gut and brain are anatomically distinct, cross-talk exists between these organs and we hypothesize that gut microbiota derived BMCs and/or EVs may have important role on the intestinal immunity and its regulation and interaction with the brain. Alteration in the gut microbiome either by infectious agents or use of licit and illicit substances has been linked to altered immune responses [108]. Moreover, gut dysbiosis promotes inflammatory and metabolic disorders [109], which may be imprinted in gut or blood derived BMCs and EVs, such as seen in EVs from saccharibacteria, formerly known as TM7, *Akkermansia muciniphila*, *Bacteroides acidifaciens* bacteria [110], or *Bacteroides fragilis*-derived EVs [111]. Thus, ES-based network may represent a link between the gut and the brain. Such an ES-mediated role may contribute to the pathogenesis of neurodegeneration, including the CD-associated neurodegeneration. 

Indeed, the gut microbiota influences gut-brain cross talk as reviewed by Van Den Elsen [112]. Multiple studies have shown that both host and microbiota derived EVs enter systemic circulation and such EVs mediate cross-talk between inflammatory pathways on both sides of the blood brain barrier (BBB) [113,114]. EVs generated by peripheral cells have been shown to contribute to various neurological deficits [115,116,117,118]. On the other hand, mesenchymal stem cell derived EVs that possess anti-inflammatory properties against myocardial inflammation, arthritis, and inflammatory lung diseases [119,120,121,122] also reduce inflammation in various CNS pathologies [123,124,125,126,127]. 

Although how EVs breach the BBB is unknown, we and others have shown that EVs facilitate release of metalloproteases, promote extracellular matrix reorganization, epithelial barrier regulation, and inflammatory cell recruitment [128]. Moreover, EVs containing immune/inflammatory and epithelial cell specific markers are elevated in the blood and intestinal lumen of patients suffering from IBD and such high EV concentrations are correlated with disease severity [129]. In this context, a causal relationship between increased number of cargo-carrying EVs in blood/intestinal tissue and enhanced intestinal permeability has been reported in IBS [93]. It is therefore clear that communication between the brain and the periphery is a bidirectional interaction as demonstrated by the entrance of blood-derived EVs into the CNS and uptake by microglia cells [103] vis a vis the detection of CNS-derived EVs in the blood [130]. Therefore, both of these cross interactions must have occurred following EV-mediated transport through the BBB.

Although how gut- or neurodegenerative-linked BMCs or EVs arise, as well as the exact mechanisms behind their ability to regulate gut-directed neurodegeneration, is still not well understood, we propose multiple possibilities based on our studies and the literature. First, ES released by intestinal cells and/or microbiota may be decorated with degradative enzymes such as matrix metalloproteinases (MMPs) or microbiota-derived ES-mediated degradation of polysaccharides and inositol polyphosphates. Second, such degradative molecules may promote the ability of ES to access and cross the epithelial barrier, gain access to systemic circulation, and reach other organs, and the brain. Third, compromised intestinal epithelial barrier and BBB may facilitate transcellular transmigration and deposition of inflammatory cargo. Alternatively, peripheral ES may use other mechanisms to reach the CNS or vice versa via paracellular transmigration, micropinocytosis, clathrin-mediated endocytosis, or caveolin-mediated endocytosis. 

## 3. Conclusions

In summary, there are several avenues for shaping the dysregulated gut homeostasis in individuals with CD, NCGS and/or neurodegeneration. In addition to GFD [62], probiotics [131], and nutraceuticals, including potentially synthetic cannabinoids, such as selective cannabinoid receptor agonists, there are other evolving [132] strategies that could shape the host microbiome towards better prognostic outcomes of associated neuropathological disorders. To narrow down these approaches to the most promising ones, preclinical trials with the rhesus macaque model of CD can help to achieve these goals. 

## Abbreviations

CD	Celiac Disease
GFD	Gluten-Free Diet
tTG2	Intestinal Tissue Transglutaminase
tTG6	Neural Tissue Transglutaminase
NCGS	Non-Celiac Gluten Sensitivity
CNS	Central Nervous System
BBB	Blood-Brain Barrier
HLA	Human leukocyte antigen
mRNA	messenger RNA
miRNA	micro RNA
tRNA	transfer RNA
dsDNA	double-stranded DNA
SIV	Simian Immunodeficiency Virus
AD	Alzheimer’s Disease
PD	Parkinson’s Disease
HD	Huntington’s Disease
ASD	Autism Spectrum Disorder
DS	Down’s Syndrome
IBD	Inflammatory Bowel Disease
IBS	Irritable Bowel Syndrome
ZO1	Zona Occludens Protein 1
PPARγ	Peroxisome proliferator activated receptor gamma
STAT3	Signal transducer and activator of transcription 3
c-MYC	MYC proto-oncogene BHLH transcription factor
NOTCH1	Notch receptor 1
KLF4	Kruppel like factor 4
NFκB	Nuclear factor kappa B
NOS2	Nitric oxide synthase 2
THC	Delta-9-Tetra-Hydrocannabinol
ES	Extracellular Structure
BMS	Biomolecular Condensate
EV	Extracellular Vesicle
FTD	Frontotemporal Dementia
ALS	Amyotrophic Lateral Sclerosis
MAPT	Microtubule-Associated Protein Tau
